# Brachial artery flow-mediated dilation and mean platelet volume in relation to diabetic retinopathy severity in type 2 diabetes: a retrospective cross-sectional study

**DOI:** 10.3389/fendo.2026.1911136

**Published:** 2026-10-22

**Authors:** Ying Zeng, Congyao Wang, Zhimin Huang, Tingting Chen, Fengjuan Yao, Wenhui Zhu

**Affiliations:** 1 Department of Ophthalmology, The First Affiliated Hospital of Sun Yat-sen University, Guangzhou, Guangdong, China; 2 Department of Endocrinology, The First Affiliated Hospital of Sun Yat-sen University, Guangzhou, Guangdong, China; 3 Department of Medical Ultrasonics, Institute for Diagnostic and Interventional Ultrasound, The First Affiliated Hospital of Sun Yat-sen University, Guangzhou, Guangdong, China

**Keywords:** carotid ultrasonography, diabetic retinopathy, endothelial function, flow-mediated dilation, mean platelet volume, type 2 diabetes mellitus

## Abstract

**Background:**

To evaluate the cross-sectional associations of brachial artery flow-mediated dilation (FMD) and mean platelet volume (MPV) with diabetic retinopathy (DR) severity in adults with type 2 diabetes mellitus (T2DM), after adjustment for age, sex, diabetes duration, glycated hemoglobin (HbA1c), and systolic blood pressure; carotid ultrasound measures were examined secondarily.

**Methods:**

This single-center, hospital-based cross-sectional study retrospectively analyzed clinical and imaging data obtained during routine inpatient care through a standardized multidisciplinary assessment pathway from 109 hospitalized adults with T2DM between June 2015 and December 2019: 46 with no DR (NDR), 36 with non-proliferative DR (NPDR), and 27 with proliferative DR (PDR). The main exploratory complete-case proportional-odds model included 86 participants (36 NDR, 30 NPDR, and 20 PDR) and simultaneously included age, sex, diabetes duration, HbA1c, systolic blood pressure, FMD, and MPV; a nephropathy-adjusted model and a ridge-penalized model were examined as sensitivity analyses.

**Results:**

Median FMD was 6.9%, 6.1%, and 5.3% in the NDR, NPDR, and PDR groups (Kruskal-Wallis P = .025), and median MPV was 9.30, 9.60, and 10.00 fL (P = .012). In the main exploratory complete-case model (N = 86), lower FMD (adjusted odds ratio [OR] per 1-percentage-point increase, 0.831; 95% CI, 0.713-0.968; P = .018) and higher MPV (adjusted OR per 1-fL increase, 1.898; 95% CI, 1.238-2.912; P = .003) were associated with greater DR severity. After additional adjustment for recorded diabetic nephropathy, the FMD estimate was attenuated (OR, 0.855; 95% CI, 0.732-0.998), whereas the MPV estimate was preserved (OR, 2.166; 95% CI, 1.356-3.458). In a ridge-penalized sensitivity model, both estimates were attenuated (FMD OR, 0.899 and 0.919; MPV OR, 1.388 and 1.289 at the minimum-loss and one-standard-error penalties, respectively).

**Conclusions:**

Lower brachial FMD and higher MPV were associated with greater DR severity in this selected hospital-based cross-sectional sample; the FMD association was sensitive to renal adjustment, and both associations, particularly that for MPV, were noticeably attenuated under penalization. These exploratory findings do not establish temporal sequence, causality, predictive performance, or clinical utility and require confirmation in larger prospective cohorts.

## 1 Introduction

Diabetic retinopathy (DR) remains a leading cause of preventable vision loss in working-age adults and is among the earliest clinically detectable microvascular complications of type 2 diabetes mellitus (T2DM) (1, 2). The global prevalence of DR among adults with diabetes is approximately 22%, and the number of affected individuals is projected to exceed 160 million by 2045 (3). Although chronic hyperglycemia is central to its pathogenesis, DR develops in the context of systemic vascular injury, oxidative stress, low-grade inflammation, and altered platelet biology (4, 5). Whether non-invasive systemic vascular and platelet-related measures are associated with DR severity after adjustment for major clinical covariates remains uncertain.

Endothelial dysfunction, characterized by reduced nitric oxide bioavailability and impaired endothelium-dependent vasodilation, accompanies microvascular and macrovascular complications of diabetes (5–7). Brachial artery FMD is a non-invasive measure of endothelium-dependent vasodilation that has been standardized in international guidance and associated with cardiovascular outcomes (8–11). T2DM is associated with lower FMD (12), and small cross-sectional studies have reported lower FMD in patients with DR. Whether FMD remains associated with ordered DR severity after adjustment for major clinical factors is less clear.

Structural carotid ultrasound, including carotid intima–media thickness (C-IMT) and plaque, provides complementary information on macrovascular disease (13). MPV is a method-dependent platelet-size index that has been associated with platelet activity and reactivity and with DR in prior studies (14–17). Previous studies have related FMD, carotid IMT, or MPV individually to the presence of DR (16, 17, 25–27); fewer data address whether these measures are associated with ordered DR severity when evaluated jointly with adjustment for clinical covariates.

In this retrospective cross-sectional study of hospitalized adults with T2DM, we therefore examined (1) whether brachial FMD, carotid ultrasound parameters, and platelet indices differed across DR severity strata and (2) whether FMD and MPV were associated with ordered DR severity after adjustment for a clinically selected set of demographic and clinical covariates. The specific contribution of this study is the joint evaluation of continuous FMD and MPV in one multivariable proportional-odds model of ordered DR severity, with carotid ultrasound measures examined secondarily.

## 2 Materials and methods

### 2.1 Design and participants

This single-center, hospital-based study was a retrospective analysis of clinical and imaging data obtained during routine inpatient care of adults with T2DM hospitalized in the Department of Endocrinology of the First Affiliated Hospital of Sun Yat-sen University between June 2015 and December 2019. During this period, the Departments of Endocrinology and Ophthalmology and the Vascular Ultrasound Unit applied a standardized collaborative assessment pathway for diabetic vascular complications, under which T2DM inpatients of the Department of Endocrinology received an inpatient ophthalmology consultation with dilated fundus examination and fundus photography, and brachial FMD and carotid ultrasonography performed routinely by one designated experienced sonographer, irrespective of cardiovascular risk profile or DR status. Potentially eligible patients were identified by a retrospective search of the institutional electronic medical record for Department of Endocrinology inpatients with T2DM who had both a documented ophthalmology consultation with DR grading and a brachial FMD examination during the study period; all patients identified by this search were screened against the eligibility criteria. Eligibility screening, data extraction, DR grouping for analysis, and all statistical analyses were performed retrospectively without additional patient contact.

Clinical assessments performed between June 2015 and May 2018 were routine clinical examinations conducted as part of standard inpatient care and were documented in the clinical record; they were not performed under a research protocol. The collaborative vascular-assessment protocol was approved by the Institutional Ethics Committee of the First Affiliated Hospital of Sun Yat-sen University on May 29, 2018 (approval no. [2018]124). The retrospective analysis of de-identified clinical and imaging records from June 2015 to December 2019 was approved by the same committee on October 31, 2025 (approval no. [2025]699), which waived the requirement for written informed consent because the analysis used de-identified retrospective data and involved no additional patient contact or intervention. The study was conducted in accordance with the Declaration of Helsinki and reported in accordance with STROBE guidance (18).

T2DM was defined according to the American Diabetes Association criteria (19). Exclusion criteria were type 1 diabetes; prior retinal photocoagulation, intravitreal pharmacotherapy, or retinal surgery; other retinal disorders; media opacity precluding fundus grading; pregnancy or lactation; heavy smoking or alcohol misuse recorded at assessment; active infection or clinically important inflammation; severe renal impairment documented as an eGFR <30 mL/min/1.73 m² or renal replacement therapy; clinically manifest peripheral arterial disease; active malignancy; and systolic blood pressure >180 mm Hg at screening. Among 129 potentially eligible patients, 20 were excluded: 2 because of systolic blood pressure >180 mm Hg at screening, 10 because the brachial FMD examination was incomplete, and 8 because of technically inadequate imaging (ungradable retinal photography in at least one eye, n=3; technically inadequate carotid ultrasonography, n=3; both, n=2). The remaining 109 participants were included in descriptive analyses; 86 complete cases were included in the main exploratory multivariable model (**Figure 1**).

**Figure 1:**
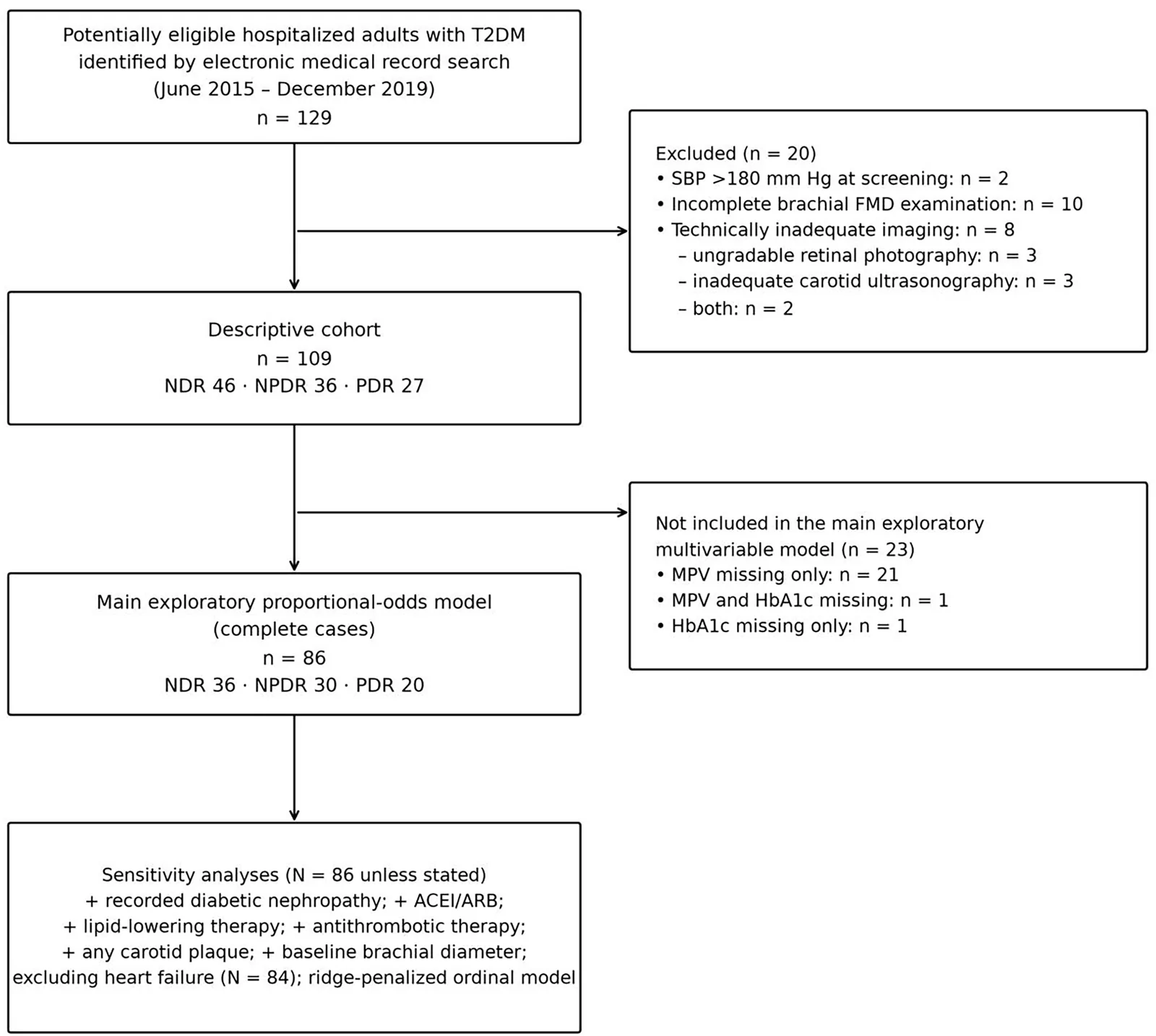
Flow diagram of participant identification, exclusion, and analysis. Patients were identified by an electronic medical record search of T2DM inpatients who completed the collaborative ophthalmology–vascular ultrasound assessment pathway between June 2015 and December 2019. FMD, flow-mediated dilation; HbA1c, glycated hemoglobin; MPV, mean platelet volume; NDR, no diabetic retinopathy; NPDR, non-proliferative diabetic retinopathy; PDR, proliferative diabetic retinopathy; SBP, systolic blood pressure.

### 2.2 Clinical and laboratory assessment

Demographic characteristics, diabetes duration, comorbidities, medication classes (ACEI/ARB, lipid-lowering, and antithrombotic therapy), and lifestyle factors were abstracted from the medical record and available questionnaires. Height, weight, waist and hip circumferences, and seated blood pressure were measured using calibrated equipment. Blood pressure was measured twice after 5 minutes of seated rest, and the mean was used. Body mass index was calculated as weight in kilograms divided by height in meters squared. Hypertension was defined by a recorded diagnosis, antihypertensive treatment, or office blood pressure ≥140/90 mm Hg. Diabetic-nephropathy status was based on the diagnosis recorded in the clinical record; participant-level eGFR and albuminuria values were not available in the final de-identified analysis dataset, so this variable may misclassify renal disease. Within the collaborative assessment pathway, brachial FMD and carotid ultrasonography were performed in one session by the same sonographer, fasting venous blood was drawn on the first morning after admission as part of routine inpatient testing, and the ophthalmology consultation took place during the same hospitalization, within a maximum interval of 2 weeks; participant-level assessment dates were not retained in the analysis dataset, so the observed intervals could not be reconstructed.

Venous blood was obtained after an overnight fast of at least 8 hours. For platelet indices, EDTA-anticoagulated whole blood was stored at 24-28 °C and analyzed within 60 minutes. All complete blood counts during the study period were performed on one hematology analyzer (Sysmex XN-20; Sysmex Corporation, Kobe, Japan); the institutional transition to a different platform occurred in 2020, after the last participant was examined. MPV is a method-dependent platelet-size index, and absolute values are not directly transferable to other analyzers. PDW was defined as the width of the platelet-size distribution curve at the 20% frequency level (fL). Laboratory measurements included HbA1c, fasting glucose, lipid measures, creatinine, blood urea nitrogen (BUN), uric acid, liver enzymes, albumin, C-reactive protein, complete blood count, platelet count, plateletcrit, MPV, and PDW. Central laboratory methods for these measurements were unchanged during the study period.

### 2.3 Ophthalmic evaluation and DR classification

All participants underwent a comprehensive ophthalmic examination after pharmacological pupil dilation, including slit-lamp biomicroscopy and indirect ophthalmoscopy, together with standardized color fundus photography obtained with a retinal camera (KOWA VX-10, Japan), providing one 45° field centered on the macula for each eye; the same camera and photographic protocol were used throughout the study period. Photographs were considered gradable when focus and clarity permitted identification of microaneurysms, illumination was adequate and even, and the macula and optic disc were fully visualized; photography was repeated in the same session when these criteria were not met for technical reasons. DR severity was graded by two experienced retinal specialists, masked to clinical and vascular-ultrasound findings, on the basis of the dilated fundus examination combined with the fundus photograph, using the International Clinical Diabetic Retinopathy Disease Severity Scale (20); when the two sources were discordant, the more severe finding determined the grade, and disagreements between graders were resolved by consensus. When the eyes differed, the participant was assigned the grade of the more severely affected eye; participants in whom a DR grade could not be assigned in either eye were excluded. Participants were classified as NDR, NPDR, or PDR. The NPDR group combined mild (n=6), moderate (n=10), and severe (n=20) NPDR because the individual subgroup sizes were too small to support a more granular ordinal outcome with the intended multivariable adjustment set; this *post hoc* grouping improved numerical stability at the cost of reduced severity resolution. Only the adjudicated grade was retained, so pre-arbitration inter-reader agreement could not be calculated. Diabetic macular edema (DME) status was not documented uniformly and is described in the **Supplementary Material** only.

### 2.4 Vascular ultrasound and FMD measurement

Vascular ultrasound examinations were performed by one experienced sonographer masked to ophthalmic findings, using a 5-12-MHz linear-array probe (GE Vivid 7; GE Healthcare); the same system, protocol, and sonographer were used throughout the study period. As part of the standard examination procedure in force at the time of measurement,participants fasted for at least 8 hours and were instructed to avoid caffeine, alcohol, smoking, vigorous exercise, and vasoactive medication before examination; adherence was checked by interview and examinations were rescheduled when restrictions were not met. After 15 minutes of supine rest in a temperature-controlled room, the right brachial artery was imaged 2–5 cm proximal to the antecubital fossa. A single-lead ECG from the ultrasound system was displayed with the B-mode image, and the end-diastolic frame was identified as the frame coincident with the R-wave peak. Diameter was measured manually on this frame using the system’s electronic calipers, and three consecutive cardiac cycles were averaged. After forearm-cuff inflation to 250 mm Hg for 5 minutes, the post-deflation recording was reviewed to identify the largest end-diastolic diameter, generally within 60–90 seconds; three consecutive cycles around that point were averaged. FMD was calculated as [(maximum diameter − baseline diameter) / baseline diameter] × 100%. The absolute diameter change was also recorded (9–11). Post-deflation Doppler flow velocity was not recorded, and formal intraobserver repeatability was not assessed.

Bilateral common carotid ultrasonography recorded common-carotid diameter, flow velocity, and far-wall IMT 10 mm proximal to the bifurcation; three measurements per side were averaged. Plaque was defined using Mannheim-consensus criteria as a focal structure protruding into the lumen by ≥0.5 mm, by ≥50% of the surrounding IMT, or with thickness ≥1.5 mm (13). Because the reproducibility of the ordinal plaque-grade coding could not be verified, analyses used binary any-plaque status only.

### 2.5 Statistical analysis

Continuous variables are summarized as median (Q1, Q3), and categorical variables as n (%) or n/N (%) when data were incomplete. Comparisons across NDR, NPDR, and PDR used the Kruskal-Wallis test for continuous variables and the chi-square test or, when any expected count was <5, the Fisher-Freeman-Halton exact test for categorical variables. These omnibus tests do not assess an ordered trend, and no trend test was performed. All P values are two-sided and unadjusted for multiplicity; because of the number of group comparisons, univariable analyses, and sensitivity analyses, P values are presented as descriptive measures of compatibility with the null hypothesis in exploratory analyses rather than as confirmatory evidence, and no fixed significance threshold was applied.

Sample size was not determined by a formal power calculation because this exploratory retrospective study included all eligible participants during the study period. Associations with ordered DR severity (NDR < NPDR < PDR) were examined using proportional-odds logistic regression. The main exploratory multivariable model simultaneously included age, sex, diabetes duration, HbA1c, systolic blood pressure, FMD, and MPV. Age, sex, diabetes duration, HbA1c, and systolic blood pressure were selected on clinical grounds for confounding control, and FMD and MPV were the exposures of interest; no dated protocol or statistical analysis plan prespecified this model. Recorded diabetic nephropathy was not included in the main adjustment set because it was a chart diagnosis whose operational definition and chronicity could not be reconstructed, participant-level eGFR and albuminuria were unavailable, and the complete-case sample supported additional terms only marginally; it was instead examined in a sensitivity model reported alongside the main model. Additional medication and carotid variables were examined one at a time in sensitivity analyses because the complete-case sample was limited. The descriptive cohort comprised 109 participants (46 NDR, 36 NPDR, and 27 PDR), whereas the main exploratory multivariable model included 86 participants (36, 30, and 20, respectively). Results are reported as ORs with 95% CIs. Given the modest analysis-specific sample, all multivariable estimates were interpreted as exploratory.

The main exploratory multivariable model included 86 complete cases (36 NDR, 30 NPDR, and 20 PDR). Twenty-three participants were excluded because of missing MPV and/or HbA1c: MPV only (n=21), MPV plus HbA1c (n=1), or HbA1c only (n=1). MPV was available for 87/109 participants and HbA1c for 107/109. Of the 22 participants with missing MPV, the complete blood count was not captured in the extracted record in 14, and in 8 the report did not include platelet indices; because all participants preceded the analyzer transition, missingness could not be related to a change in laboratory platform. Complete cases were compared with excluded participants using the Mann-Whitney U test and Fisher exact tests (**Supplementary Table S2**).

Analyses were performed in Python 3.11 using pandas 3.0.5, NumPy 2.4.6, SciPy 1.17.1, and statsmodels 0.15.0. The ordinal model was fitted with statsmodels OrderedModel (logit link) using the BFGS optimizer. The proportional-odds assumption was evaluated with a Brant-type test: the two cumulative binary logistic regressions (NDR vs NPDR+PDR; NDR+NPDR vs PDR) were fitted, and equality of their coefficients was tested with a Wald statistic using the model-based cross-threshold covariance, following the procedure implemented in the R package brant (version 0.3-0), with global and variable-specific statistics. Functional form was assessed by adding, one at a time, a truncated-power restricted cubic spline with three knots at the 10th, 50th, and 90th percentiles (one nonlinear basis term) for each continuous predictor and testing nonlinearity with a 1-df likelihood-ratio test; these were diagnostic checks of the ordinal model, not prediction analyses. Collinearity was assessed using variance-inflation factors, and influence was examined with leave-one-out refits. Sensitivity analyses added recorded diabetic-nephropathy status, ACEI/ARB use, lipid-lowering therapy, antithrombotic therapy, any carotid plaque, or baseline brachial diameter one at a time and separately excluded participants with heart failure. To assess stability under shrinkage, a ridge-penalized cumulative-logit proportional-odds model with the same seven predictors was fitted to the same 86 complete cases, with all seven predictors standardized and the thresholds unpenalized. The penalty was selected by fixed-seed, five-times-repeated, stratified 10-fold cross-validation on the held-out multinomial negative log-likelihood. Results are reported at the penalty that minimized the mean cross-validated loss (minimum-loss penalty) and at the largest penalty whose mean loss was within one standard error of that minimum (one-standard-error penalty), with coefficients back-transformed to the original units; the selected penalty values are reported with the estimates. Penalized estimates are presented as sensitivity diagnostics rather than confirmatory inference.

## 3 Results

### 3.1 Participant characteristics

The descriptive cohort included 109 Department of Endocrinology inpatients examined between June 2015 and December 2019: 46 with NDR, 36 with NPDR, and 27 with PDR (**Figure 1**). Baseline characteristics are shown in **Table 1**.The omnibus comparisons across the NDR, NPDR, and PDR groups yielded P values of.104 to.973 for age, sex distribution, body mass index, systolic and diastolic blood pressure, diabetes duration, and HbA1c (**Table 2**), P = .168 for hypertension (47.8%, 58.3%, and 70.4%), and P = .117 for ACEI/ARB use (23.9%, 44.4%, and 40.7%); these results do not establish that the groups were comparable with respect to these characteristics. Recorded diabetic nephropathy (2.2%, 16.7%, and 29.6%; P = .001) and heart failure (0%, 0%, and 11.1%; P = .014) differed across groups. Comparisons between complete cases (N = 86) and the 23 participants excluded from the main model yielded P values of.230 to.807 for DR distribution, age, sex, FMD, and recorded nephropathy, whereas excluded participants had higher BUN and creatinine (**Supplementary Table S2**).

**Table 1:** Baseline clinical and demographic characteristics by DR severity.

Variable	Total (N = 109)	NDR (n=46)	NPDR (n=36)	PDR (n=27)	P value
Male sex	39 (35.8)	15 (32.6)	16 (44.4)	8 (29.6)	.402
Urban residence	81/106 (76.4)	39 (84.8)	22/34 (64.7)	20/26 (76.9)	.112
Age, y	59 (50, 66)	59 (45, 65)	58 (53, 66)	59 (54, 72)	.184
Diabetes duration, y	9.0 (2.0, 14.0)	5.5 (2.0, 11.8)	10.0 (1.8, 14.2)	10.0 (6.5, 15.0)	.261
BMI, kg/m²	23.7 (21.3, 25.4)	24.0 (21.3, 26.1)	24.0 (23.0, 25.4)	23.0 (21.0, 25.0)	.620
SBP, mm Hg	129 (117, 138)	126 (116, 135)	130 (114, 137)	132 (122, 142)	.104
DBP, mm Hg	80 (70, 86)	80 (67, 86)	80 (69, 87)	79 (74, 82)	.973
Waist–hip ratio (n=108)	0.92 (0.89, 0.98)	0.91 (0.88, 0.95)	0.93 (0.90, 0.99)	0.96 (0.88, 0.98)	.286
Hypertension	62 (56.9)	22 (47.8)	21 (58.3)	19 (70.4)	.168
ACEI/ARB use	38 (34.9)	11 (23.9)	16 (44.4)	11 (40.7)	.117
Lipid-lowering therapy	49 (45.0)	20 (43.5)	14 (38.9)	15 (55.6)	.406
Diabetic nephropathy (recorded)	15 (13.8)	1 (2.2)	6 (16.7)	8 (29.6)	.001
Heart failure	3 (2.8)	0 (0.0)	0 (0.0)	3 (11.1)	.014
Antithrombotic therapy	29 (26.6)	10 (21.7)	10 (27.8)	9 (33.3)	.546

ACEI, angiotensin-converting-enzyme inhibitor; ARB, angiotensin-receptor blocker; BMI, body mass index; DBP, diastolic blood pressure; DR, diabetic retinopathy; NDR, no diabetic retinopathy; NPDR, non-proliferative diabetic retinopathy; PDR, proliferative diabetic retinopathy; SBP, systolic blood pressure. Continuous variables are presented as median (Q1, Q3); categorical variables as number (percentage). P values are from the Kruskal-Wallis test for continuous variables and the chi-square test or Fisher-Freeman-Halton exact test (when any expected count was <5) for categorical variables; all P values are two-sided, unadjusted for multiplicity, and descriptive. For variables with missing data, available-case denominators are reported as n/N. Hypertension was defined as a recorded diagnosis, antihypertensive treatment, or office blood pressure ≥140/90 mm Hg.

**Table 2:** Laboratory variables by DR severity.

Variable	Total (N = 109)	NDR (n=46)	NPDR (n=36)	PDR (n=27)	P value
HbA1c, % (n=107)	8.8 (7.2, 10.7)	8.8 (7.3, 11.0)	8.8 (7.1, 10.6)	8.0 (7.2, 10.5)	.907
Fasting glucose, mmol/L	7.4 (6.0, 10.3)	7.2 (5.9, 10.1)	7.7 (6.2, 11.4)	7.4 (5.9, 11.8)	.579
CRP, mg/L (n=92)	1.27 (0.68, 3.80)	1.33 (0.78, 3.94)	1.08 (0.48, 5.07)	1.30 (0.80, 2.75)	.870
WBC, ×10^9^/L (n=87)	7.26 (5.62, 8.23)	6.82 (5.67, 7.50)	7.42 (5.55, 8.90)	7.73 (5.79, 8.34)	.205
Platelet count, ×10^9^/L (n=95)	235 (196, 286)	230 (196, 278)	232 (194, 260)	260 (207, 314)	.308
Plateletcrit, % (n=87)	0.220 (0.190, 0.270)	0.218 (0.180, 0.250)	0.220 (0.190, 0.270)	0.230 (0.210, 0.310)	.370
MPV, fL (n=87)	9.62 (8.88, 10.30)	9.30 (8.42, 9.93)	9.60 (9.03, 10.53)	10.00 (9.60, 10.60)	.012
PDW, fL (n=87)	12.60 (10.80, 16.43)	16.34 (11.28, 16.87)	11.95 (10.15, 14.07)	12.00 (11.30, 14.00)	.052
Hemoglobin, g/L (n=92)	134 (121, 144)	136 (126, 142)	138 (123, 146)	126 (117, 140)	.237
Albumin, g/L (n=84)	40.5 (38.2, 43.0)	40.3 (38.9, 43.0)	39.6 (37.4, 42.5)	41.2 (39.8, 43.0)	.555
ALT, U/L (n=85)	20 (15, 28)	18 (14, 28)	22 (15, 28)	24 (19, 30)	.344
AST, U/L (n=85)	22 (18, 25)	20 (18, 24)	20 (17, 25)	23 (20, 26)	.327
Total cholesterol, mmol/L	4.9 (4.2, 5.9)	4.9 (4.3, 5.7)	4.8 (4.1, 6.1)	5.0 (3.9, 5.8)	.678
Triglycerides, mmol/L	1.31 (0.98, 2.30)	1.12 (0.88, 1.98)	1.45 (1.15, 2.42)	1.42 (1.20, 2.50)	.053
HDL-C, mmol/L	1.13 (0.98, 1.33)	1.17 (0.99, 1.33)	1.10 (0.94, 1.31)	1.13 (1.03, 1.34)	.380
LDL-C, mmol/L	3.15 (2.51, 3.73)	3.15 (2.60, 3.50)	3.00 (2.41, 4.03)	3.22 (2.26, 3.59)	.554
TG/HDL-C ratio	1.20 (0.82, 2.20)	0.94 (0.69, 2.08)	1.39 (0.99, 2.21)	1.26 (0.92, 2.04)	.043
BUN, mmol/L	5.2 (4.3, 6.4)	4.5 (3.9, 5.2)	5.7 (4.9, 6.7)	5.6 (4.8, 6.6)	.001
Creatinine, µmol/L	62 (53, 78)	60 (54, 70)	66 (52, 83)	73 (58, 88)	.075
Uric acid, µmol/L	355 (291, 401)	348 (271, 386)	355 (318, 398)	382 (309, 414)	.321

ALT, alanine aminotransferase; AST, aspartate aminotransferase; BUN, blood urea nitrogen; CRP, C-reactive protein; DR, diabetic retinopathy; HbA1c, glycated hemoglobin; HDL-C, high-density lipoprotein cholesterol; LDL-C, low-density lipoprotein cholesterol; MPV, mean platelet volume; NDR, no diabetic retinopathy; NPDR, non-proliferative DR; PDR, proliferative DR; PDW, platelet distribution width; TG, triglycerides; WBC, white blood cell count. Values are median (Q1, Q3); P values are from the Kruskal-Wallis test (omnibus; not a test of ordered trend). All complete blood counts were analyzed on one hematology analyzer (Sysmex XN-20); PDW is the platelet-size distribution width at the 20% frequency level. Valid sample sizes are shown in row labels when fewer than 109 observations were available; group-specific valid sample sizes (NDR/NPDR/PDR) were: HbA1c, %, 46/35/26; CRP, mg/L, 45/25/22; WBC, ×10⁹/L, 37/30/20; Platelet count, ×10⁹/L, 40/32/23; Plateletcrit, %, 36/30/21; MPV, fL, 36/30/21; PDW, fL, 36/30/21; Hemoglobin, g/L, 38/32/22; Albumin, g/L, 37/28/19; ALT, U/L, 38/28/19; AST, U/L, 38/28/19.

### 3.2 Laboratory findings

Laboratory findings are summarized in **Table 2**. BUN differed across DR groups (4.5, 5.7, and 5.6 mmol/L; P = .001), without a monotonic pattern. The TG/HDL-C ratio also differed (0.94, 1.39, and 1.26; P = .043). Median MPV was 9.30, 9.60, and 10.00 fL in NDR, NPDR, and PDR (P = .012); the omnibus test does not assess an ordered trend. PDW medians were 16.35, 11.95, and 12.00 fL (P = .052); PDW was not included in the multivariable model.

### 3.3 Vascular ultrasound findings

Brachial and carotid ultrasound findings are presented in **Table 3**. Median baseline and maximal brachial diameters were 3.62, 3.65, and 3.46 mm (P = .731) and 3.92, 3.81, and 3.58 mm (P = .462) in the NDR, NPDR, and PDR groups, respectively. Median absolute diameter change was 0.27, 0.21, and 0.19 mm (P = .008), and median FMD was 6.9%, 6.1%, and 5.3% in NDR, NPDR, and PDR (P = .025). Baseline diameter, maximal diameter, and diameter change were available for 108 participants (one NPDR participant had a recorded FMD value only), and FMD for all 109. FMD was analyzed only as a continuous measure.

**Table 3:** Brachial and carotid ultrasound parameters by DR severity.

Variable	Total (N = 109)	NDR (n=46)	NPDR (n=36)	PDR (n=27)	P value
Brachial baseline diameter, mm (n=108)	3.56 (3.16, 3.89)	3.62 (3.19, 3.87)	3.65 (3.16, 3.96)	3.46 (3.13, 3.83)	.731
Brachial maximal diameter, mm (n=108)	3.80 (3.40, 4.10)	3.92 (3.48, 4.19)	3.81 (3.46, 4.16)	3.58 (3.36, 3.96)	.462
ΔDiameter, mm (n=108)	0.23 (0.17, 0.30)	0.27 (0.20, 0.31)	0.21 (0.15, 0.28)	0.19 (0.13, 0.29)	.008
FMD, %	6.5 (4.7, 8.4)	6.9 (6.0, 9.4)	6.1 (4.0, 8.3)	5.3 (4.0, 8.0)	.025
Left CCA diameter, mm (n=108)	6.40 (5.90, 6.90)	6.30 (5.83, 6.80)	6.40 (5.95, 6.90)	6.40 (6.10, 6.95)	.868
LIMT, mm (n=108)	0.80 (0.60, 0.90)	0.70 (0.60, 0.80)	0.80 (0.60, 0.90)	0.80 (0.70, 1.00)	.044
Right CCA diameter, mm (n=108)	6.60 (6.10, 7.10)	6.50 (5.95, 6.79)	6.50 (6.10, 7.10)	7.00 (6.22, 7.60)	.069
RIMT, mm (n=108)	0.80 (0.66, 0.92)	0.70 (0.60, 0.82)	0.80 (0.70, 0.88)	0.89 (0.80, 1.01)	.005
Any carotid plaque	61/108 (56.5)	20 (43.5)	22/35 (62.9)	19 (70.4)	.053

CCA, common carotid artery; DR, diabetic retinopathy; FMD, flow-mediated dilation; LIMT, left carotid intima-media thickness; NDR, no diabetic retinopathy; NPDR, non-proliferative DR; PDR, proliferative DR; RIMT, right carotid intima-media thickness. Values are median (Q1, Q3) or n/N (%). One NPDR participant had a recorded FMD value but no retained baseline diameter, maximal diameter, or diameter change (n=108 for these rows); carotid measures and plaque status were unavailable for one NPDR participant (n=108). P values are from the Kruskal-Wallis or chi-square test and are descriptive. DME status is reported in the **Supplementary Material** only because of extreme differential missingness.

Right and left carotid IMT differed across DR groups (P = .005 and P = .044, respectively). Any carotid plaque was present in 20/46 (43.5%) NDR, 22/35 (62.9%) NPDR, and 19/27 (70.4%) PDR participants (P = .053). These carotid comparisons were secondary and exploratory. DME status was documented for only 51 participants, with extreme differential missingness across groups, and is described in the **Supplementary Material** without inferential comparison.

### 3.4 Multivariable associations with DR severity

Complete univariable proportional-odds results, including analysis-specific sample sizes and missing counts, are provided in **Supplementary Table S1**. The main exploratory seven-variable proportional-odds model and the nephropathy-adjusted sensitivity model are shown in **Table 4**.

**Table 4:** Main exploratory proportional-odds model for DR severity (Complete-case N = 86; NDR < NPDR < PDR) and nephropathy-adjusted sensitivity model.

Variable	Main model: adjusted OR	95% CI	P value	Nephropathy-adjusted model: adjusted OR	95% CI	P value
Age (per 1 y)	1.032	0.993-1.073	.110	1.035	0.996-1.076	.076
Sex (female vs male)	0.897	0.353-2.284	.820	0.922	0.357-2.384	.867
Diabetes duration (per 1 y)	1.003	0.947-1.064	.907	0.994	0.937-1.055	.850
HbA1c (per 1%)	0.990	0.842-1.163	.899	0.917	0.769-1.094	.338
SBP (per 1 mm Hg)	1.027	0.998-1.056	.065	1.020	0.991-1.050	.174
FMD (per 1 percentage point)	0.831	0.713-0.968	.018	0.855	0.732-0.998	.047
MPV (per 1 fL)	1.898	1.238-2.912	.003	2.166	1.356-3.458	.001
Recorded diabetic nephropathy (yes vs no)	—	—	—	5.834	1.498-22.715	.011

DR, diabetic retinopathy; FMD, flow-mediated dilation; HbA1c, glycated hemoglobin; MPV, mean platelet volume; OR, odds ratio. The outcome was ordered as NDR < NPDR < PDR. Both models included 86 complete cases (36 NDR, 30 NPDR, and 20 PDR); all variables were entered simultaneously. The seven-variable model was selected on clinical grounds and was not prospectively prespecified. Global Brant chi-square=2.724, 7 df; P = .909. P values are two-sided, unadjusted for multiplicity, and exploratory.

In the 86-participant complete-case model, lower FMD (adjusted OR per 1-percentage-point increase, 0.831; 95% CI, 0.713-0.968; P = .018) and higher MPV (adjusted OR per 1-fL increase, 1.898; 95% CI, 1.238-2.912; P = .003) were associated with greater DR severity. The global Brant test did not indicate violation of the proportional-odds assumption(chi-square=2.724, 7 df; P = .909; variable-specific P = .276-.931). No continuous term showed detectable nonlinearity (1-df likelihood-ratio P = .401-.727); VIFs ranged from 1.040 to 1.179, and all 86 leave-one-out refits converged without reversal of the FMD or MPV association (FMD OR range 0.783-0.843; MPV OR range 1.823-2.186).

When recorded diabetic nephropathy was added to the model (N = 86), nephropathy was associated with greater DR severity (OR, 5.834; 95% CI, 1.498-22.715; P = .011), the FMD estimate was attenuated with an upper confidence limit approaching 1.00 (OR, 0.855; 95% CI, 0.732-0.998; P = .047), and the MPV estimate was preserved (OR, 2.166; 95% CI, 1.356-3.458; P = .001) (**Table 4**). Adding ACEI/ARB use, lipid-lowering therapy, antithrombotic therapy, any carotid plaque, or baseline brachial diameter one at a time, or excluding the two participants with heart failure, yielded FMD ORs of 0.770-0.861 and MPV ORs of 1.867-1.911 (**Supplementary Table S3**). In the ridge-penalized sensitivity model, both associations retained their direction but were noticeably attenuated (**Supplementary Table S4**). At the minimum-loss penalty (λ=11.620), the FMD OR was 0.899 per 1-percentage-point increase and the MPV OR was 1.388 per 1-fL increase; at the one-standard-error penalty (λ=20.153), the corresponding ORs were 0.919 and 1.289. Relative to the unpenalized model (FMD OR, 0.831; MPV OR, 1.898), the FMD log-odds coefficient was reduced by approximately 43% and 54% and the MPV coefficient by approximately 49% and 60% at the two penalties, respectively.

## 4 Discussion

In this hospital-based retrospective cross-sectional study of adults with T2DM, two principal observations emerged. First, median FMD was lower in groups with more severe DR, and FMD was inversely associated with ordered DR severity after adjustment for age, sex, diabetes duration, HbA1c, systolic blood pressure, and MPV; this association was attenuated, with a confidence interval approaching the null, after additional adjustment for recorded diabetic nephropathy, and was also attenuated under ridge penalization. Second, higher MPV was associated with greater DR severity in the same complete-case model; this association was preserved after additional adjustment for recorded diabetic nephropathy but was noticeably attenuated in the ridge-penalized sensitivity analysis. These findings should be interpreted cautiously because of the modest, single-center sample, missing data, and incomplete control of potential confounding.

An association between lower FMD and more severe DR is biologically plausible. Chronic hyperglycemia may promote oxidative stress, advanced glycation, protein-kinase-C activation, reduced nitric oxide bioavailability, pericyte loss, and disruption of the blood-retinal barrier (5, 21–23). Our findings are consistent with prior reports of impaired FMD in T2DM (12, 24) and smaller cross-sectional studies linking lower FMD with retinopathy (25–27). The present analysis adds an evaluation of continuous FMD against ordered DR severity with simultaneous adjustment for MPV and a clinically selected covariate set, while showing that the FMD estimate is sensitive to renal adjustment.

MPV is a method-dependent platelet-size index associated with platelet activity and reactivity, rather than a direct measure of *in-vivo* platelet activation. Larger platelets may express more glycoprotein IIb/IIIa, produce more thromboxane A2, and aggregate more readily (28, 29). Prior studies and meta-analyses have reported higher MPV in DR, particularly PDR (15–17, 30, 31).Tissue studies have also shown platelet accumulation in the diabetic retinal vasculature following endothelial cell death (32) and an increased prevalence of platelet–fibrin microthrombi in retinal capillaries obtained post mortem from diabetic donors (33); these findings provide a biological context for a link between platelet characteristics and DR, although our data cannot address this mechanism. The present cross-sectional association supports further prospective study of MPV, but it does not establish a diagnostic cutoff, predictive value, or clinical utility.

The ridge-penalized sensitivity analysis further qualifies the magnitude of both associations. Under penalization, both associations retained their direction but were noticeably attenuated: the FMD OR moved toward the null from 0.831 to 0.899 and 0.919, and the MPV OR decreased from 1.898 to 1.388 and 1.289, at the minimum-loss and one-standard-error penalties, respectively. The attenuation was more pronounced for MPV, whose log-odds coefficient was reduced by approximately 49% and 60%, compared with approximately 43% and 54% for FMD. Penalized estimates are shrunk toward the null by construction and should not be regarded as corrected effect estimates; nevertheless, the degree of attenuation indicates that the unpenalized estimates from this small complete-case sample, which included only 20 participants with PDR, are imprecise and may overstate the strength of both associations. The unpenalized ORs, particularly that for MPV, should therefore not be interpreted as reliable estimates of effect size.

BUN and recorded diabetic-nephropathy status differed across DR groups, and adding recorded nephropathy to the model materially attenuated the FMD estimate. Retinopathy and nephropathy share several risk factors and pathophysiologic processes (34–40). However, participant-level eGFR and albuminuria were unavailable, the recorded nephropathy definition could not be reconstructed fully, and this variable may misclassify renal disease; the nephropathy-adjusted model therefore does not fully control renal confounding, particularly given the large difference in recorded nephropathy prevalence across DR categories. The renal findings should be interpreted as exploratory confounding assessments rather than independent mechanistic evidence.

## Limitations

Several limitations should be acknowledged. First, this was a single-center, hospital-based study with a modest sample. The cohort was defined by completion of a collaborative ophthalmology–vascular ultrasound assessment pathway; T2DM inpatients who did not receive an ophthalmology consultation or an FMD examination were not captured, and indications for hospitalization were not recorded systematically and may have been associated with both DR severity and vascular function. These features introduce selection bias and limit generalizability to community-based populations. Because patients with prior retinal photocoagulation, intravitreal treatment, or retinal surgery were excluded, the PDR group represents a selected subset of patients not previously treated at the time of assessment and may not be representative of the broader PDR population.

Second, the cross-sectional design precludes temporal or causal inference. Lower FMD and higher MPV may have preceded advanced DR, resulted from other diabetic complications, or been influenced by treatment and behavioral changes after DR diagnosis. Participant-level assessment dates were not retained, so the intervals between retinal examination, blood sampling, and vascular ultrasonography could not be quantified; changes in glycemia, blood pressure, hydration, inflammation, and medication exposure during hospitalization could have affected FMD and platelet indices between nonsynchronous assessments.

Third, the main adjusted analysis included only 86 complete cases because MPV and/or HbA1c were missing in 23 participants. Excluded participants had higher BUN and creatinine than complete cases, and because the absence of detected differences in DR distribution, age, sex, and FMD does not establish comparability, complete-case selection bias cannot be excluded. The analysis-specific sample limited the number of covariates that could be included and increased the risks of imprecision, residual confounding, and chance findings across multiple secondary comparisons; no multiplicity adjustment was applied, and all analyses are exploratory. In the ridge-penalized sensitivity analysis, both associations retained their direction but were noticeably attenuated, particularly the MPV association (OR, 1.388 and 1.289 at the minimum-loss and one-standard-error penalties vs 1.898 in the unpenalized model); the magnitudes of the unpenalized estimates are therefore uncertain and may be overstated.

Fourth, confounding was incompletely controlled. Recorded diabetic-nephropathy status may have misclassified renal disease because participant-level eGFR, albuminuria, and chronicity data were unavailable, and the nephropathy-adjusted model—in which the FMD estimate was attenuated—therefore does not fully control renal confounding. Medication sensitivity analyses added broad classes one at a time and cannot adequately control simultaneous or time-varying treatment effects; individual drugs, doses, durations, treatment changes, and SGLT2-inhibitor and GLP-1-receptor-agonist use were unavailable, so residual medication confounding remains likely. Current HbA1c was available for 107 of 109 participants and was included as a covariate in the main exploratory multivariable model. However, longitudinal historical HbA1c measurements were not captured, and a single current value cannot represent cumulative glycemic exposure or metabolic memory over the years preceding the development of DR. Diabetes duration was included as a covariate, but it does not fully substitute for cumulative glycemic exposure. Residual confounding by historical glycemia therefore cannot be excluded. Some retrospectively recorded exclusion criteria, including heavy smoking, alcohol misuse, and clinically important inflammation, lacked uniformly retained operational definitions.

Fifth, measurement limitations apply to both exposures and the outcome. Although all FMD examinations were performed by one experienced sonographer using manual caliper measurements, formal intraobserver repeatability was not assessed; the absence of repeated-measure agreement data, such as an intraclass correlation coefficient or coefficient of variation, introduces additional measurement uncertainty beyond that addressed by the use of a single operator. Post-deflation Doppler flow velocity was unavailable, preventing quantification of reactive hyperemia and the shear-rate stimulus; lower measured FMD may therefore reflect not only reduced endothelial responsiveness but also between-participant differences in the magnitude of the post-occlusion shear stimulus. MPV is a method-dependent index, and although all samples were analyzed on one analyzer, absolute values are not transferable across platforms. DR grading combined a dilated fundus examination with one macula-centered 45° photograph per eye; standardized multi-field or widefield imaging was not available, so some peripheral lesions may have been under-documented and residual misclassification of the ordinal DR outcome cannot be excluded. Because graders were masked to vascular and laboratory data, differential misclassification with respect to FMD and MPV is less likely; however, for a three-level ordinal outcome, the direction of any resulting bias cannot be assumed, and the estimates should be interpreted with this uncertainty in mind. Only the adjudicated DR grade was retained, preventing calculation of pre-arbitration inter-reader agreement.

Finally, the NPDR group combined mild, moderate, and severe NPDR, reducing severity resolution. Participant-level eGFR and intraocular-pressure values were unavailable. DME status was highly incomplete and differentially available across DR groups and was therefore described only in the **Supplementary Material** without inferential comparison.

## Conclusions

In this hospital-based retrospective cross-sectional study of adults with T2DM examined between June 2015 and December 2019, lower brachial FMD and higher MPV were associated with greater DR severity in a main exploratory complete-case proportional-odds model. The FMD association was attenuated after adjustment for recorded diabetic nephropathy, whereas the MPV association was not; under ridge penalization, both associations retained their direction but were noticeably attenuated, particularly the MPV association, so their magnitudes remain uncertain. Carotid and renal measures also differed across DR groups in exploratory analyses. These findings identify cross-sectional associations rather than causal, predictive, or clinically actionable relationships and require confirmation in larger prospective multicenter cohorts.

## Data Availability

The datasets generated and analyzed during the current study are available from the corresponding author upon reasonable request, subject to institutional ethics committee approval and participant privacy protection. Requests to access the datasets should be directed to Wenhui Zhu, zhuwh@mail.sysu.edu.cn.
