# Supplementary material for: Brachial artery flow-mediated dilation and mean platelet volume in relation to diabetic retinopathy severity in type 2 diabetes: a retrospective cross-sectional study
Source: Front Endocrinol (Lausanne). 2026 Oct 22;17:1911136. doi: 10.3389/fendo.2026.1911136 (PMC13735542; doi:10.3389/fendo.2026.1911136)
Supplement: Supplementary file 1 [file SupplementaryFile1.docx]

SUPPLEMENTARY MATERIAL

# Technical summary

Main exploratory result. In the source-verified complete-case proportional-odds model (N=86), lower flow-mediated dilation (FMD) and higher mean platelet volume (MPV) were associated with greater diabetic retinopathy (DR) severity after adjustment. The adjusted odds ratio (OR) was 0.831 per 1-percentage-point increase in FMD (95% CI 0.713-0.968; P = .018) and 1.898 per 1-fL increase in MPV (95% CI 1.238-2.912; P = .003).

**Model validity.** The global Brant test did not indicate violation of the proportional-odds assumption (chi-square = 2.724, 7 df; P = .909). No assessed continuous term showed detectable nonlinearity, variance-inflation factors ranged from 1.040 to 1.179, and all 86 leave-one-out refits converged without reversal of the FMD or MPV association.

**Interpretation.** These cross-sectional, single-center findings are exploratory. Complete-case analysis, modest group sizes, multiple secondary comparisons, and substantial missingness for selected ophthalmic variables limit causal and predictive interpretation.

# 1. Scope and source-data verification

The final DR cohort comprised 109 participants: 46 without diabetic retinopathy (NDR), 36 with non-proliferative diabetic retinopathy (NPDR), and 27 with proliferative diabetic retinopathy (PDR). Analyses used the final de-identified standardized dataset. One platelet-index record showing a shifted triplet was reconciled against source documentation before analysis; the verified values were plateletcrit (PCT) 0.296, MPV 8.54. The source workbook was preserved, and the verified values were used in every result reported here.

The main exploratory model included 86 complete cases (36 NDR, 30 NPDR, and 20 PDR). Twenty-three participants were excluded from that model because of missing MPV and/or HbA1c: MPV only (n=21), MPV plus HbA1c (n=1), or HbA1c only (n=1).

# 2. Statistical analysis

Ordinal DR severity (NDR < NPDR < PDR) was modeled using proportional-odds logistic regression. The main exploratory adjustment set comprised FMD, MPV, age, sex, diabetes duration, HbA1c, and systolic blood pressure (SBP). ORs greater than 1 indicate higher cumulative odds of belonging to a more severe DR category. All analyses were complete-case and all P values were two-sided. No multiplicity adjustment was applied; secondary analyses are therefore descriptive and hypothesis-generating.

The proportional-odds assumption was evaluated with global and variable-specific Brant Wald tests. Continuous-variable functional form was assessed by adding the nonlinear component of a three-knot restricted cubic spline (10th, 50th, and 90th percentiles) to the full model one variable at a time. Collinearity was assessed with variance-inflation factors (VIFs). Influence was evaluated by 86 leave-one-out refits and standardized coefficient changes using |DFBETA| > 2/sqrt(N) as a screening threshold. Cumulative binary models were also checked for convergence and complete separation.

Sensitivity analyses added diabetic nephropathy, ACE inhibitor/angiotensin receptor blocker (ACEI/ARB) use, lipid-lowering therapy, antithrombotic therapy, any carotid plaque, or baseline brachial diameter one at a time, and separately excluded participants with heart failure (Supplementary Table S3). Prediction-performance analyses (ROC curves, calibration, and apparent in-sample AUC comparisons) were not retained because they lacked internal or external validation. Analyses were performed in Python 3.12.13 using pandas 3.0.5, NumPy 2.5.2, SciPy 1.18.0, and statsmodels 0.14.6.

# 3. Final analytic results

## Lower FMD and higher MPV were associated with greater DR severity after adjustment

The seven-predictor proportional-odds model converged (log likelihood -79.531; AIC 177.062; BIC 199.151). The complete model is shown below so that the adjustment set and all coefficient estimates can be audited directly.

Main exploratory proportional-odds model for DR severity (N=86)

| Variable | Adjusted OR | 95% CI | P |
| --- | --- | --- | --- |
| FMD (per 1 percentage point) | 0.831 | 0.713-0.968 | .018 |
| MPV (per 1 fL) | 1.898 | 1.238-2.912 | .003 |
| Age (per year) | 1.032 | 0.993-1.073 | .110 |
| Female vs male | 0.897 | 0.353-2.284 | .820 |
| Diabetes duration (per year) | 1.003 | 0.947-1.064 | .907 |
| HbA1c (per 1%) | 0.990 | 0.842-1.163 | .899 |
| SBP (per 1 mmHg) | 1.027 | 0.998-1.056 | .065 |

Outcome order: NDR < NPDR < PDR. OR>1 indicates greater cumulative odds of more severe DR. Sex was coded as female versus male.

## MPV distributions across DR categories

MPV differed across DR categories (Kruskal-Wallis P=.012). Because an equivalent definition and unit for PDW could not be verified across the two hematology platforms, pooled PDW descriptive and univariable analyses were removed.

Platelet-index distributions by DR category

| Index | NDR median (IQR); n/N | NPDR median (IQR); n/N | PDR median (IQR); n/N | P |
| --- | --- | --- | --- | --- |
| PCT | 0.2185 (0.1798-0.2500); 36/46 | 0.2200 (0.1900-0.2700); 30/36 | 0.2300 (0.2100-0.3100); 21/27 | .370 |
| MPV | 9.300 (8.418-9.925); 36/46 | 9.600 (9.025-10.525); 30/36 | 10.000 (9.600-10.600); 21/27 | .012 |

P values are from Kruskal-Wallis tests. PCT, plateletcrit; MPV, mean platelet volume.

## Model assumptions and influence checks did not identify a material violation

Variable-specific model diagnostics

| Variable | Brant P | Nonlinearity P | VIF |
| --- | --- | --- | --- |
| FMD (per 1 percentage point) | .695 | .659 | 1.143 |
| MPV (per 1 fL) | .488 | .543 | 1.040 |
| Age (per year) | .758 | .412 | 1.179 |
| Female vs male | .276 | Not applicable | 1.100 |
| Diabetes duration (per year) | .902 | .431 | 1.113 |
| HbA1c (per 1%) | .724 | .727 | 1.065 |
| SBP (per 1 mmHg) | .931 | .401 | 1.096 |

Global Brant chi-square = 2.724 with 7 df (P = .909). Nonlinearity was assessed one continuous variable at a time; sex was not assessed. VIFs include an intercept in the auxiliary regressions.

All 86 leave-one-out models converged. The FMD OR ranged from 0.783 to 0.843, and the MPV OR ranged from 1.823 to 2.186; neither association changed direction. At |DFBETA|>0.216, 4 observations were flagged for FMD and 6 for MPV. Both cumulative binary models converged, with no complete separation detected.

## Sensitivity analyses were directionally consistent, with expected imprecision

Adding recorded diabetic nephropathy attenuated the FMD estimate (OR 0.855; 95% CI 0.732-0.998) and preserved the MPV estimate (OR 2.166; 95% CI 1.356-3.458). Adding ACEI/ARB use, lipid-lowering therapy, antithrombotic therapy, any carotid plaque, or baseline brachial diameter one at a time, or excluding the two participants with heart failure, yielded FMD ORs of 0.770-0.861 and MPV ORs of 1.867-1.911. All sensitivity models are reported in Supplementary Table S3.

## Denominator reconciliation changed interpretation of selected secondary findings

Reconciled denominators for selected variables

| Variable | Overall | NDR | NPDR | PDR | Note |
| --- | --- | --- | --- | --- | --- |
| Urban residence | 81/106 (76.4%) | 39/46 | 22/34 | 20/26 | 3 missing |
| Any carotid plaque | 61/108 (56.5%) | 20/46 | 22/35 | 19/27 | 1 NPDR value missing |
| Diabetic macular edema | 38/51 (74.5%) | 0/1 | 16/25 | 22/25 | 58 missing; descriptive only |
| Main model complete cases | 86/109 (78.9%) | 36/46 | 30/36 | 20/27 | Analysis-specific N |

Diabetic macular edema was evaluable in only one NDR participant. The highly differential missingness precludes stable group inference; these counts are descriptive only.

# 4. Missing data and analysis-specific denominators

The main exploratory model was complete for 86/109 participants. Across the core variables used in the descriptive tables, 48/109 participants had at least one missing value and 61 were fully complete. Missingness was concentrated in laboratory variables measured in subsets and in selected ophthalmic variables.

Variable-level missing-data summary for key secondary measures

| Variable | Non-missing | Missing | Missing (%) |
| --- | --- | --- | --- |
| HbA1c | 107 | 2 | 1.8% |
| Platelet count | 95 | 14 | 12.8% |
| Plateletcrit | 87 | 22 | 20.2% |
| Mean platelet volume | 87 | 22 | 20.2% |
| Platelet distribution width | 87 | 22 | 20.2% |
| Any carotid plaque | 108 | 1 | 0.9% |
| Diabetic macular edema | 51 | 58 | 53.2% |
| High myopia | 14 | 95 | 87.2% |

Counts are based on the final DR cohort (N=109). Variables without missing data that were included in the main exploratory model were age, sex, diabetes duration, SBP, and FMD; HbA1c had 2 missing values and MPV had 22.

# 5. Interpretation and reporting constraints

The final analysis supports an exploratory association between lower FMD, higher MPV, and greater DR severity. It does not establish temporal direction, causality, mediation, or validated predictive performance. The modest complete-case sample, 20 PDR cases in the main exploratory model, and wide intervals in several sensitivity models limit precision.

DME should be reported only as a descriptive subset with explicit n/N denominators. No inference should be based on apparent training-sample discrimination or calibration because those analyses were not internally validated.

Pre-analytical details for platelet indices (analyzer model, anticoagulant, storage conditions, and time from phlebotomy to analysis) were not available in the analysis dataset and should be reported from laboratory source documentation in the main manuscript.

# Supplementary Table S1. Univariable proportional-odds models

Each row represents a separate univariable proportional-odds model for ordinal DR severity. Results are reported in the reported variable order and were not selected by statistical significance.

## Clinical

| Variable / comparison | N | Missing | OR | 95% CI | P |
| --- | --- | --- | --- | --- | --- |
| Female vs male | 109 | 0 | 0.982 | 0.479-2.011 | .960 |
| Rural vs urban residence | 106 | 3 | 1.608 | 0.721-3.589 | .246 |
| Age (per year) | 109 | 0 | 1.034 | 1.003-1.065 | .030 |
| Diabetes duration (per year) | 109 | 0 | 1.036 | 0.989-1.086 | .135 |
| BMI (per 1 kg/m²) | 109 | 0 | 0.969 | 0.888-1.058 | .485 |
| SBP (per 10 mmHg) | 109 | 0 | 1.198 | 0.963-1.491 | .106 |
| DBP (per 10 mmHg) | 109 | 0 | 1.059 | 0.779-1.440 | .713 |
| Hypertension grade (per category) | 109 | 0 | 1.407 | 1.027-1.929 | .034 |
| Diabetic nephropathy (present vs absent) | 109 | 0 | 5.576 | 1.951-15.934 | .001 |
| ACEI/ARB use (yes vs no/other) | 109 | 0 | 1.899 | 0.918-3.927 | .084 |
| Lipid-lowering therapy (yes vs no) | 109 | 0 | 1.321 | 0.654-2.668 | .438 |
| Heart failure (yes vs no) | 109 | 0 | NE | NE | NE |
| Alcohol use (yes vs no) | 109 | 0 | 1.506 | 0.181-12.504 | .704 |

NE, not estimable. Heart failure occurred only in PDR (3 cases), producing separation. Antithrombotic therapy, for which one participant had a nonstandard source code, was examined only as an added covariate in the main exploratory model (Supplementary Table S3).

## Laboratory

| Variable / comparison | N | Missing | OR | 95% CI | P |
| --- | --- | --- | --- | --- | --- |
| HbA1c (per 1%) | 107 | 2 | 0.977 | 0.855-1.117 | .734 |
| C-reactive protein (per 1 mg/L) | 92 | 17 | 0.996 | 0.957-1.037 | .858 |
| Platelet count (per 10 x10^9/L) | 95 | 14 | 1.035 | 0.983-1.089 | .191 |
| Plateletcrit (per 0.1 recorded unit) | 87 | 22 | 1.448 | 0.802-2.615 | .220 |
| MPV (per 1 fL) | 87 | 22 | 1.655 | 1.159-2.364 | .006 |
| White blood cell count (per 1 x10^9/L) | 87 | 22 | 1.204 | 1.014-1.431 | .034 |
| Red blood cell count (per 1 x10^12/L) | 87 | 22 | 1.088 | 0.575-2.055 | .796 |
| Lymphocyte count (per 1 x10^9/L) | 87 | 22 | 1.056 | 0.580-1.921 | .860 |
| Albumin (per 1 g/L) | 84 | 25 | 0.977 | 0.918-1.039 | .461 |
| HALP score (per 10 units) | 95 | 14 | 0.930 | 0.820-1.055 | .261 |
| ALT (per 10 U/L) | 85 | 24 | 1.045 | 0.876-1.246 | .626 |
| AST (per 10 U/L) | 85 | 24 | 0.948 | 0.720-1.247 | .702 |
| AST/ALT ratio (per 1 unit) | 85 | 24 | 0.338 | 0.107-1.070 | .065 |
| ALP (per 10 U/L) | 84 | 25 | 1.052 | 0.901-1.228 | .523 |
| Hemoglobin (per 10 g/L) | 92 | 17 | 0.898 | 0.727-1.109 | .319 |
| Lipoprotein(a) (per 10 recorded units) | 92 | 17 | 0.990 | 0.977-1.004 | .146 |
| Total cholesterol (per 1 mmol/L) | 109 | 0 | 0.893 | 0.685-1.165 | .404 |
| Triglycerides (per 1 mmol/L) | 109 | 0 | 1.155 | 0.861-1.549 | .336 |
| HDL cholesterol (per 1 mmol/L) | 109 | 0 | 0.641 | 0.159-2.575 | .531 |
| LDL cholesterol (per 1 mmol/L) | 109 | 0 | 0.818 | 0.569-1.177 | .279 |
| Fasting glucose (per 1 mmol/L) | 109 | 0 | 1.038 | 0.951-1.133 | .405 |
| Blood urea nitrogen (per 1 mmol/L) | 109 | 0 | 1.163 | 0.983-1.375 | .078 |
| Creatinine (per 10 umol/L) | 109 | 0 | 1.170 | 1.019-1.343 | .026 |
| Uric acid (per 10 umol/L) | 109 | 0 | 1.019 | 0.979-1.061 | .352 |
| TG/HDL ratio (per 1 unit) | 109 | 0 | 1.092 | 0.813-1.466 | .559 |
| Uric acid/HDL-C ratio (per 1 recorded unit) | 109 | 0 | 1.001 | 0.998-1.004 | .431 |

The urinary protein/creatinine field was omitted because source coding and units could not be verified. Platelet-index estimates use the source-verified PCT and MPV values. ALT, alanine aminotransferase; AST, aspartate aminotransferase; ALP, alkaline phosphatase; HDL-C, high-density lipoprotein cholesterol; HALP, hemoglobin-albumin-lymphocyte-platelet score. Variables labeled per recorded unit retain the scale stored in the standardized analysis dataset.

## Vascular

| Variable / comparison | N | Missing | OR | 95% CI | P |
| --- | --- | --- | --- | --- | --- |
| Any carotid plaque (yes vs no) | 108 | 1 | 2.424 | 1.169-5.022 | .017 |
| Baseline brachial diameter (per 0.1 mm) | 108 | 1 | 0.995 | 0.936-1.058 | .872 |
| Brachial IMT (per 0.1 mm) | 109 | 0 | 1.217 | 0.903-1.640 | .197 |
| Baseline flow velocity (per 10 recorded units) | 108 | 1 | 1.164 | 0.993-1.364 | .062 |
| Maximum brachial diameter (per 0.1 mm) | 108 | 1 | 0.980 | 0.921-1.042 | .518 |
| Peak flow velocity (per 10 recorded units) | 108 | 1 | 1.022 | 0.940-1.112 | .606 |
| Diameter change (per 0.1 mm) | 108 | 1 | 0.578 | 0.391-0.853 | .006 |
| 30-second flow velocity (per 10 recorded units) | 107 | 2 | 1.012 | 0.905-1.132 | .830 |
| FMD (per 1 percentage point) | 109 | 0 | 0.857 | 0.762-0.964 | .010 |
| Left carotid diameter (per 0.1 mm) | 108 | 1 | 0.994 | 0.952-1.038 | .781 |
| Left carotid IMT (per 0.1 mm) | 108 | 1 | 1.216 | 1.021-1.449 | .028 |
| Left bifurcation IMT (per 0.1 mm) | 108 | 1 | 1.088 | 0.939-1.259 | .261 |
| Left carotid peak velocity (per 10 recorded units) | 108 | 1 | 0.884 | 0.734-1.064 | .192 |
| Right carotid diameter (per 0.1 mm) | 108 | 1 | 1.052 | 1.004-1.103 | .032 |
| Right carotid IMT (per 0.1 mm) | 108 | 1 | 1.367 | 1.127-1.659 | .002 |
| Right bifurcation IMT (per 0.1 mm) | 106 | 3 | 1.183 | 1.021-1.370 | .025 |
| Right carotid peak velocity (per 10 recorded units) | 107 | 2 | 0.854 | 0.691-1.057 | .147 |

The ordinal plaque-grade model was omitted because category definitions could not be verified; binary any-plaque status was retained. IMT, intima-media thickness. Variables labeled per recorded unit retain the scale stored in the standardized analysis dataset. All secondary P values are exploratory and unadjusted for multiplicity.

# 6. Round-2 additional analyses and source-field audit

The final corrected patient-level workbook contained no laboratory, retinal, FMD, carotid, admission, or discharge dates and no screening/exclusion log. Therefore, pre/post-2020 analyses, MPV missingness by calendar period, assessment-interval summaries, and a modality-specific decomposition of the 20 pre-cohort exclusions could not be performed without retrieval of source records. Worksheet row order was not used as a proxy for time.

MPV was available for 87/109 (79.8%) participants: 36/46 (78.3%) NDR, 30/36 (83.3%) NPDR, and 21/27 (77.8%) PDR. The Fisher-Freeman-Halton P value was .866; this group-based comparison does not answer calendar-period missingness.

## Supplementary Table S2. Complete cases versus participants excluded from the main model

| Characteristic | Complete cases (n=86) | Excluded (n=23) | Exploratory P |
| --- | --- | --- | --- |
| DR category, n (%) | NDR 36/86 (41.9%); NPDR 30/86 (34.9%); PDR 20/86 (23.3%) | NDR 10/23 (43.5%); NPDR 6/23 (26.1%); PDR 7/23 (30.4%) | 0.664 |
| Age, years | 58 (50, 66); n=86 | 62 (53.5, 69.5); n=23 | 0.230 |
| Diabetes duration, years | 8 (2, 13); n=86 | 12 (5.5, 15.5); n=23 | 0.061 |
| HbA1c, % | 8.6 (7.05, 10.6); n=86 | 9.2 (7.5, 11.2); n=21 | 0.638 |
| Systolic blood pressure, mm Hg | 127 (116, 140); n=86 | 131 (126, 136); n=23 | 0.705 |
| FMD, % | 6.7 (4.55, 8.47); n=86 | 5.8 (5.15, 7.5); n=23 | 0.355 |
| Blood urea nitrogen | 5.1 (4, 6.07); n=86 | 5.7 (4.6, 7.45); n=23 | 0.027 |
| Creatinine | 60.5 (52, 78); n=86 | 71 (61.5, 84.5); n=23 | 0.034 |
| Female sex | 56/86 (65.1%) | 14/23 (60.9%) | 0.807 |
| Recorded diabetic nephropathy | 13/86 (15.1%) | 2/23 (8.7%) | 0.733 |
| ACEI/ARB use | 29/86 (33.7%) | 9/23 (39.1%) | 0.631 |

Continuous variables are median (Q1, Q3) with analysis-specific n; comparisons used two-sided Mann-Whitney tests. Categorical comparisons used Fisher exact or Fisher-Freeman-Halton tests. P values are descriptive and unadjusted for multiplicity; lack of a detected difference does not establish an ignorable complete-case mechanism.

## Supplementary Table S3. Sensitivity analyses based on the seven-predictor model

| Analysis | N | FMD OR (95% CI); P | MPV OR (95% CI); P | Added term OR (95% CI); P |
| --- | --- | --- | --- | --- |
| Add diabetic nephropathy | 86 | 0.855 (0.732-0.998); P = .047 | 2.166 (1.356-3.458); P = .001 | 5.834 (1.498-22.715); P = .011 |
| Exclude heart failure | 84 | 0.822 (0.703-0.962); P = .015 | 1.890 (1.229-2.906); P = .004 | - |
| Add ACEI/ARB use | 86 | 0.861 (0.735-1.008); P = .064 | 1.911 (1.244-2.937); P = .003 | 2.120 (0.790-5.687); P = .136 |
| Add lipid-lowering therapy | 86 | 0.827 (0.709-0.966); P = .016 | 1.881 (1.227-2.883); P = .004 | 1.503 (0.632-3.577); P = .357 |
| Add antithrombotic therapy | 86 | 0.832 (0.713-0.970); P = .019 | 1.880 (1.211-2.919); P = .005 | 1.099 (0.388-3.113); P = .860 |
| Add any carotid plaque | 86 | 0.848 (0.720-0.998); P = .047 | 1.880 (1.225-2.885); P = .004 | 1.397 (0.534-3.654); P = .495 |
| Add baseline brachial diameter (per 0.1 mm) | 86 | 0.770 (0.646-0.919); P = .004 | 1.867 (1.219-2.860); P = .004 | 0.871 (0.766-0.990); P = .035 |

Each sensitivity model retained FMD, MPV, age, sex, diabetes duration, HbA1c, and SBP and used the 86 complete cases, except the heart-failure exclusion (N=84). Codings follow Table 1 of the main text: recorded diabetic nephropathy and antithrombotic therapy denote any recorded diagnosis or use, ACEI/ARB use denotes antihypertensive-medication category 1, and baseline brachial diameter is entered per 0.1 mm. One complete case had a nonstandard antithrombotic source code and was classified as a user; classifying this participant as a non-user gave an FMD OR of 0.831 (0.713-0.969) and an MPV OR of 1.884 (1.210-2.932). Estimates are exploratory. The wide interval for diabetic nephropathy reflects sparse data.

## Supplementary Table S4. Ridge-penalized proportional-odds sensitivity analysis compared with the unpenalized main exploratory model (complete cases, N=86)

| Variable | Unpenalized OR | 95% CI | Minimum-loss OR (λ=11.620) | Approx. 95% interval | 1-SE OR (λ=20.153) | Approx. 95% interval |
| --- | --- | --- | --- | --- | --- | --- |
| FMD (per 1 percentage point) | 0.831 | 0.713-0.968 | 0.899 | 0.828-0.977 | 0.919 | 0.861-0.981 |
| MPV (per 1 fL) | 1.898 | 1.238-2.912 | 1.388 | 1.125-1.713 | 1.289 | 1.095-1.517 |
| Age (per year) | 1.032 | 0.993-1.073 | 1.019 | 0.997-1.041 | 1.016 | 0.998-1.033 |
| Female vs male | 0.897 | 0.353-2.284 | 0.871 | 0.496-1.529 | 0.886 | 0.566-1.389 |
| Diabetes duration (per year) | 1.003 | 0.947-1.064 | 1.008 | 0.973-1.045 | 1.008 | 0.979-1.037 |
| HbA1c (per 1%) | 0.990 | 0.842-1.163 | 0.983 | 0.889-1.087 | 0.982 | 0.906-1.065 |
| SBP (per 1 mm Hg) | 1.027 | 0.998-1.056 | 1.016 | 0.999-1.033 | 1.013 | 0.999-1.026 |

Outcome order: NDR < NPDR < PDR; all models used the same 86 complete cases (36 NDR, 30 NPDR, and 20 PDR). Unpenalized estimates are from the main exploratory model (Table 4 of the main text). All seven predictors were standardized (population standard deviation) and the two ordinal thresholds were not penalized; the penalty (λ/2)Σβj² on the standardized coefficients was added to the negative log-likelihood summed over participants. Lambda was selected by fixed-seed five-times-repeated stratified 10-fold cross-validation using held-out multinomial negative log likelihood. The minimum-loss lambda was 11.620; the one-standard-error lambda was 20.153. Penalized coefficients were back-transformed to the original units. Relative to the unpenalized model, the log-odds coefficient was reduced by 42.9% (minimum-loss penalty) and 54.5% (one-standard-error penalty) for FMD and by 48.8% and 60.4% for MPV (1 − βridge/βunpenalized, unrounded coefficients). Intervals use the approximate ridge sandwich covariance and are sensitivity diagnostics rather than confirmatory penalized-likelihood inference.

## Supplementary Table S5. Brachial-measurement denominator reconciliation

| Measure | Valid N | Missing | NDR valid | NPDR valid | PDR valid |
| --- | --- | --- | --- | --- | --- |
| Baseline diameter | 108 | 1 | 46 | 35 | 27 |
| Maximum diameter | 108 | 1 | 46 | 35 | 27 |
| Diameter change | 108 | 1 | 46 | 35 | 27 |
| FMD | 109 | 0 | 46 | 36 | 27 |
| 30-second velocity | 107 | 2 | 45 | 35 | 27 |

One NPDR record lacked baseline diameter, maximum diameter, and diameter change while retaining a recorded FMD value. Thus component analyses used N=108 and FMD used N=109. The de-identified workbook contains no stable participant identifier; source-record identification requires the authors' original crosswalk.

## Supplementary Table S6. ACEI/ARB use by DR category

| Overall | NDR | NPDR | PDR | Exact P |
| --- | --- | --- | --- | --- |
| 38/109 (34.9%) | 11/46 (23.9%) | 16/36 (44.4%) | 11/27 (40.7%) | 0.116 |

ACEI/ARB was coded as antihypertensive-medication category 1 in the source workbook. The P value is from a two-sided Fisher-Freeman-Halton exact test and is exploratory; Table 1 of the main text reports the chi-square P value (.117) for the same comparison, in line with the test-selection rule described in the main text.
